# An increased response to experimental muscle pain is related to psychological status in women with chronic non-traumatic neck-shoulder pain

**DOI:** 10.1186/1471-2474-12-230

**Published:** 2011-10-12

**Authors:** Anna Sjörs, Britt Larsson, Ann L Persson, Björn Gerdle

**Affiliations:** 1 Rehabilitation Medicine, Department of Clinical and Experimental Medicine, Linköping University, SE-581 85 Linköping, Sweden; 2 The Institute of Stress Medicine, Carl Skottsbergs gata 22B, SE-41319 Gothenburg, Sweden; 3 Pain and Rehabilitation Centre, University Hospital, SE-581 85 Linköping, Sweden; 4 Rehabilitation and Research Centre for Torture Victims, P.O. Box 2107, 1014 Copenhagen K, Denmark

**Keywords:** Quantitative sensory testing, trapezius myalgia, muscle, pain, hypersensitivity, centralization, pressure pain thresholds, pain drawing, pain intensity, questionnaire

## Abstract

**Background:**

Neck-shoulder pain conditions, e.g., chronic trapezius myalgia, have been associated with sensory disturbances such as increased sensitivity to experimentally induced pain. This study investigated pain sensitivity in terms of bilateral pressure pain thresholds over the trapezius and tibialis anterior muscles and pain responses after a unilateral hypertonic saline infusion into the right legs tibialis anterior muscle and related those parameters to intensity and area size of the clinical pain and to psychological factors (sleeping problems, depression, anxiety, catastrophizing and fear-avoidance).

**Methods:**

Nineteen women with chronic non-traumatic neck-shoulder pain but without simultaneous anatomically widespread clinical pain (NSP) and 30 age-matched pain-free female control subjects (CON) participated in the study.

**Results:**

NSP had lower pressure pain thresholds over the trapezius and over the tibialis anterior muscles and experienced hypertonic saline-evoked pain in the tibialis anterior muscle to be significantly more intense and locally more widespread than CON. More intense symptoms of anxiety and depression together with a higher disability level were associated with increased pain responses to experimental pain induction and a larger area size of the clinical neck-shoulder pain at its worst.

**Conclusion:**

These results indicate that central mechanisms e.g., central sensitization and altered descending control, are involved in chronic neck-shoulder pain since sensory hypersensitivity was found in areas distant to the site of clinical pain. Psychological status was found to interact with the perception, intensity, duration and distribution of induced pain (hypertonic saline) together with the spreading of clinical pain. The duration and intensity of pain correlated negatively with pressure pain thresholds.

## Background

Neck shoulder pain remains a major problem in work tasks with high exposure to awkward working positions, repetitive movements and movements with high precision demands. The trapezius muscle is considered particularly affected. The prevalence of chronic neck-shoulder pain appears to be higher in women than in men [1,2]. It causes high socioeconomic costs and significant loss of quality of life for the individual [3]. Because of limited knowledge of the mechanisms involved in transition from acute to chronic pain, attempts to develop effective treatments have had limited success. The clinical manifestations of chronic pain conditions include both somatic (e.g., sensory disturbances, facilitated pain responses in association with movements, tense muscles with hyperalgesia for mechanical pressure/manual palpation) and psychological symptoms (e.g., sleeping problems, anxiety, and depressive symptoms).

Sensory hypersensitivity (central sensitization is sometimes used as a synonym while others use central sensitization as a term for specific mechanisms in the central nervous system (CNS)) is a common feature of several chronic neck-shoulder pain conditions, particularly those with higher levels of pain intensity and disability [4].. At the clinical examination, this can be manifested as increased sensitivity to manual palpation (i.e., pressure), but increased sensitivity to other sensory modalities, e.g., heat or cold, have also been described [5-7]. Hypersensitivity to mechanical pressure or thermal pain is sometimes confined to the neck-shoulder area but may also be present in remote pain-free areas, even though the clinical routine examination does not reveal clinical anatomical widespread pain and/or generalized hyperalgesia for different types of stimuli [5,8-13]. Widespread deep tissue hyperalgesia has been found in patients with fibromyalgia, tension-type headache, whiplash associated disorders (WAD), idiopathic neck pain, epicondylalgia, low back pain, pelvic pain syndrome, and osteoarthritis [8,13-21]. It is generally acknowledged that the presence of widespread sensory hypersensitivity provides indication of augmented central pain processing mechanisms [4,21]. Peripheral and central sensitization and alterations in descending inhibition mechanisms of nociception have been suggested as three of the underlying mechanisms of chronic musculoskeletal pain in general [22]. It the context of muscle pain it has been suggested that neurobiological sensitization operating at somatic, cognitive and behavioral levels may increase the prevalence of e.g., sleeping problems, anxiety and depressive symptoms [23-25]. Another explanation may be that such symptoms are secondary consequences of living with chronic pain.

Pain induction in an anatomical region distant from the clinical pain region is a common strategy to investigate signs of central sensitization and/or alterations in descending inhibition of neural activity and nociception at the spinal cord level. Assessments of pain sensitivity in deep tissue of non-painful regions of the body may be of importance for better understanding of the development of widespread hypersensitivity. *Pressure pain thresholds (PPTs) using algometry *have been used extensively to map mechanical sensitivity of mainly deep tissues such as muscles. Another modality (i.e., chemical) of the pain sensitivity of muscle can be investigated using the *intramuscular hypertonic saline model *with the opportunity to assess both aspects of sensitization and referred pain [18,26,27]. The hypertonic saline model has been used extensively to characterize the sensory and motor effects involved in muscle pain, as the quality of the induced pain is comparable to acute clinical muscle pain and shows both localized and referred pain characteristics [28]. The anatomical spreading of experimentally induced muscle pain seems to alter in chronic musculoskeletal pain conditions; for example, patients with fibromyalgia experience stronger pain and larger primary and referred pain areas after hypertonic saline-evoked muscle pain compared with pain-free controls [19]. Such manifestations were present in the lower limb muscles, where these patients typically do not experience ongoing pain. Extended referred pain areas from the tibialis anterior muscle have also been found in patients with chronic WAD [18,29].

Both algometry and pain induction using the intramuscular saline model are psychophysical tests; i.e., an objective stimuli but a subjectively reported response by the tested subject. Noxious psychophysical tests require cooperation from the subject and attention, concentration, motivation and mood can reasonably affect the reports of the subjects tested [30]. A bio-psycho-social model [31,32] is preferred in clinical management of chronic pain since a blend of factors - neurobiological, psychological, coping styles, and contextual factors - contributes to the development and maintenance of chronic pain [33-38]. Moreover, psychological factors, e.g., anxiety, depressive symptoms and fear, appear to play prominent roles in maladaptive responses to pain and in pain perpetuation [32,36,39,40]. Hence, it is reasonable to assume that the psychological status can influence the reports of pain thresholds during psychophysical tests in chronic pain conditions.

Chronic WAD has been relatively extensively investigated concerning spreading of hyperalgesia as mentioned above. Studies of how widespread sensory hypersensitivity is in *non-traumatic *neck-shoulder pain disorders, e.g., chronic neck-shoulder pain, are, however, sparse and inconclusive [9,10,41] and psychological aspects have not been extensively investigated in relation to pain responses to sensory tests in these patients.

The aim of this explorative study was to further investigate signs of sensory hypersensitivity, in terms of lowered PPTs and more intense responses to painful hypertonic saline infusion, and the possible relationships to different psychological factors (sleeping problems, depression, anxiety, catastrophizing, and fear-avoidance beliefs) in women with chronic neck-shoulder pain, and various extent of regional pain, compared with healthy controls.

## Methods

### Subjects

The details concerning the recruitment of subjects have been reported elsewhere [42-44]; here is given a summary.

In order to recruit subjects with trapezius myalgia (denoted NSP), the medical reports of former female outward patients who had been referred to the multidisciplinary Pain and Rehabilitation Centre at Linköping University Hospital due to: neck myalgia and with the international classification of diseases (ICD) number M79.1, or cervicalgia ICD number M 54.2, or cervico-brachial syndrome ICD number M 53.1 and with no other diagnosis were identified. Invitation letters with information about the study were sent to 220 former patients. Those who volunteered to participate were contacted by telephone and 24 of them were invited to be examined by a standardized clinical neck and shoulder examination and to complete the Nordic Ministry Council Questionnaire (NMCQ) [45], which was used to survey their present pain.

Eligible subjects for the standardized clinical examination were those women who reported pain in the descending region of the trapezius muscle during the last seven days and reported neck and shoulder pain more than 90 days over the last 12 months. Moreover, subjects should not report pain during the last seven days from more than three body regions according to the NMCQ.

The standardised clinical examination [46] was performed to ensure that the subjects met the above criteria and that the subjects also fulfilled the criteria for trapezius myalgia. This examination included questions about pain intensity and location of pain, tiredness and stiffness in the neck-shoulder region on the day of examination, and in addition physical tests including; range of motion and tightness of muscles, pressure pain threshold and sensitivity, muscle strength and palpation of tender points. The examiner was a physician (BL), specialized in occupational medicine. The examiner was not blinded to if the subject was healthy or had pain.

The following exclusion criteria were used: 1) chronic widespread pain according to the Manchester definition [47], i. e., pain from more than two sections of two contralateral limbs and the axial skeleton present for at least three months, 2) signs of tendinitis or joint affections in the shoulders at the clinical examination, 3) prior neck trauma (according to the report of the subject), 4) rheumatoid arthritis or other systemic diseases, 5) neurological diseases, 6) metabolic diseases, 7) fibromyalgia syndrome (determined by tender point examination and pain drawing according to the ACR criteria of 1990 [48].

The diagnosis trapezius myalgia was set if the findings neck pain, tightness of the trapezius muscle (i.e., a feeling of stiffness in the descending region of the trapezius muscle was reported by the subject at examination of lateral flexion of the head) and palpable tender parts in the trapezius muscle were all included. The cervical spine was to have normal or only slightly decreased range of motion. The examination protocol allowed the examiner to identify and exclude the subjects with pain in the trapezius region that was most likely referred from painful tendons or nerve compressions in the neck and shoulder area.

Through these procedures and criteria nineteen women with chronic neck-shoulder pain, fulfilling the diagnostic criteria of trapezius myalgia (NSP) were recruited for the study (mean age: 40 years (range: 28-48 years); mean height: 168 cm (range: 160-176 cm); mean weight: 73 kg (range: 49-97 kg)). The median chronic pain duration in NSP was 120 months (range 36-273 months). The majority (n = 17) of the patients in the NSP group worked 100% or part-time. One patient was 100% on sick-leave and one received compensation for unemployment. Eighteen of these NSP subjects were subsequently included in an experimental study of repetitive work and psychosocial stress (see [42-44]).

Thirty age matched healthy women with no neck/shoulder pain, recruited via advertisements in daily newspapers, comprised the control group (denoted CON, mean age: 40 years (range: 26-50 years); mean height: 168 cm (range: 159-176 cm); mean weight: 67 kg (range: 51-90 kg)). The controls were assessed using a brief version of the clinical examination. An exclusion criterion, in addition to the above mentioned, was the presence of pain in the neck-shoulder region for more than 2-3 days during the previous 12 months.

All subjects gave their written informed consent and the study was approved by the Linköping University Ethics Committee (Dnr M46-07).

### Procedure

At the first visit those who volunteered to participate were clinically examined (see above) and if they were assessed suitable for the study, the subjects were scheduled for a second visit, 1-2 weeks later, which comprised different measurements of pain sensitivity. At the time of the clinical examination (i.e., first visit) they were given a questionnaire to be completed at home and instructed to bring it back the next visit. All measurements during the second visit were performed by the same research nurse, using the same sequence of testing, starting with pressure pain thresholds, followed by hypertonic saline infusion for all subjects.

### Pressure pain thresholds

Pressure pain thresholds (PPT) were measured with an electronic algometer (Somedic Production, Stockholm, Sweden) at three points located in the right and left trapezius muscles, respectively; T1 (medial), T2 (middle), and T3 (lateral) and at one reference point over the right and left tibialis anterior muscles of the lower leg. Two measurements were recorded at each site with approximately a 1-minute pause between the measurements. The measurements were made in a fixed order, starting with the medialpoint over the right trapezius muscle, continuing with the corresponding points on the left side, followed by the reference points over the right and left tibialis anterior muscles. The contact area of the algometer probe tip was 1 cm^2 ^and was covered with 2-mm-thick rubber to minimize irritation of the skin. The pressure was applied perpendicularly to the skin at a rate of 40 kPa/s. A scale on the display helped the investigator to keep the rate of the pressure increase fixed. The participants were instructed to depress a handheld switch at their first perception of pain, i.e., when the sensation of "pressure" changed to "pain or discomfort", at which point the application of pressure ceased. The registered pressure threshold measured in kilo Pascals (kPa) was then frozen on the display unit. To avoid bruising due to tissue damage a cut-off point was set at 600 kPa. The mean values of T1+T2+T3 were calculated and presented as results.

A test trial, on a single point over the rhomboid muscles bilaterally, was performed to familiarize the participant with the procedure. All measurements were carried out by the same research nurse. The technique has been found to have a satisfactory repeatability [49].

### Induced muscle pain

Experimental muscle pain was induced by injection of 0.5 ml sterile hypertonic saline (5.8%) into the tibialis anterior muscle of the right lower leg with the subjects placed comfortably in a sitting position for the injection. The needle was inserted into the deep mid-portion of the tibialis anterior muscle. The bolus was injected during a 20 s period using a computer-controlled syringe pump (IVAC, model 770). A tube (IVAC G30303, extension set with polyethylene inner line) was connected from the syringe to a stainless disposable needle (27 G, 19 mm) inserted into the muscle. Pain intensity was rated by the patients using a 0-10 cm electronic visual analogue scale (VAS) immediately following the injection and every 5 s until pain was no longer reported. Time to pain onset, peak pain intensity, time to peak pain (the point in time when maximum pain was first reached), mean pain intensity, and area under the VAS-time graph were extracted.

Participants documented the area of pain on a front and back view body template. The pain drawings were subsequently digitized and the pain areas were estimated (Quantify Image, K:L:O:N:K, Sorø, Denmark). Pain areas that were isolated from the area of local pain caused by the saline injection were denoted as referred pain areas. Proximal pain was defined as a pain area that spread proximal to the knee joint. The total size of the pain area and the areas of referred pain were calculated.

### Clinical pain drawings

The patients with chronic neck-shoulder pain (NSP) shaded-in their painful areas on three separate body templates (front and back views)[50]; one drawing for their 'current pain', one for their 'least pain' during the last week, and one for their 'worst pain' during the last week. The areas on the pain drawings were measured in square millimeters and calculated as percentage of the total body template area using a commercial software program (Quantify One; K:L:O:N:K, Sorø, Denmark), a method that has been shown to be reliable for quantifying pain drawings [51,52].

### Questionnaire

The questionnaire comprised pain intensity ratings and instruments concerning various aspects of pain and psychological factors.

***Pain intensity ***regarding clinical pain in the neck and shoulder regions was rated on a 0-100 mm paper visual analogue scale (VAS) with the defined end points "no pain" and "worst possible pain". All the questions regarding pain concerned the previous 30 days.

***Karolinska Sleep Questionnaire (KSQ) ***was used to assess sleep disturbances and fatigue over the past six months [53]. KSQ comprises 15 items rated on a 5-point scale and three indices: "awakening problems" (denoted KSQ-Aw), "daytime sleepiness" (denoted KSQ-Dst), and "sleep disturbances" (denoted KSW-Sd) were calculated from 12 of the 15 items.

***Hospital Anxiety and Depression Scale (HADS) ***is a self-rating scale in which the severity of anxiety and depressive symptoms is rated on a 4-point scale. Seven questions are related to anxiety and seven to depression, each with a score range of 0-21. A score of 7 or less indicates a non-case, a score of 8-10 a doubtful case, and 11 or more a definite case [54].

***Anxiety Sensitivity Index (ASI) ***is a 16-item self-report questionnaire. Each item asks about the amount of fear the participant experiences in regard to bodily sensations commonly associated with anxiety. Participants are asked to rate each item on a 5-point Likert-like scale ranging from *very little *(0) to *very much *(4). The ratings on the 16 items are summed for a total ranging from 0 to 64 [55].

***Pain Anxiety Symptoms Scale-20 (PASS-20) ***measures fear and anxiety responses specific to pain [56,57]. The PASS-20 has four 5-item subscales that measure avoidance, fearful thinking, cognitive anxiety and physiological responses to pain. Participants rate each item on a 6-point scale ranging from *never *(0) to *always *(5).

***Pain Catastrophizing Scale (PCS)***, a 13-item self-report measure designed to assess catastrophic thoughts or feelings accompanying the experience of pain. Respondents are asked to reflect on past painful experiences and to indicate the degree to which each of the 13 thoughts or feelings are experienced when in pain. The questionnaire uses a 5-point scale ranging from 0 (not at all) to 4 (all the time) and, in this study, we used the total sum score [58,59].

***Fear-Avoidance Beliefs Questionnaire (FABQ) ***was used to assess fear-avoidance beliefs. The FABQ is a 16-item self-report questionnaire aimed at quantifying the beliefs of how work and physical activity affect pain and whether they should be avoided. The two subscales, fear-avoidance beliefs for work (FABQwork) and fear-avoidance beliefs for physical activity (FABQphysical), are scored on a 7-point Likert scale (0-6) ranging from 'strongly disagree' to 'strongly agree', where higher sum scores indicate stronger fear-avoidance beliefs [60].

***Pain Disability Index (PDI)***, a 7-item self-report instrument based on a 10-point scale that assesses perception of the specific impact of pain on disability that may preclude normal or desired performance of a wide range of functions, such as family and social activities, sex, work, life-support (sleeping, breathing, eating), and activities of daily living [61,62].

### Statistics and Data analysis

Statistical analyses were performed in SPSS version 17 (SPSS Inc.) and SIMCA -P+ version 12.0 (Umetrics Inc). Statistical significance was defined as p < .05. Differences between groups (NSP and CON) in pain sensitivity measurements and questionnaire scores were analyzed using Mann-Whitney U-tests. Spearman correlation coefficients (rho) were calculated to analyze possible relationships between PPT and hypertonic saline-evoked pain and between pain sensitivity measurements and questionnaire scores. In the Tables the median and range values are given for each variable.

Principal component analysis (PCA), using SIMCA-P+, was used to investigate multivariate correlations between pain variables and psychological variables within the NSP-group. PCA can be viewed as a multivariate correlation analysis. Related methods such as factor analysis (i.e., including rotation of the factor solution) assume a high subject-to-variables ratio is present (5-10). But such requirements are not required for the PCA included in the SIMCA-P+ package. Furthermore a cross validation technique was used to identify nontrivial principal components (PC). This method keeps part of the data out from the model development to assess the predictive power of the model and was used to test the significance of the components. Hence, this validation technique increases the stability of the results. Such validation is not implemented in other common statistical packages e.g., SPSS. Variables loading upon the same component are correlated and variables with high loadings but with different signs are negatively correlated. Variables with absolute loadings >0.20 and that had a 95% confidence interval not equal to zero were considered significant. Significant variables with high loadings (positive or negative) are more important for the component under consideration than variables with lower absolute loadings. Variables of a certain principal component with significant loadings and the same sign are positively correlated while loadings of variables with different signs denote negative correlations. The obtained principal components are per definition not correlated and are arranged in decreasing order with respect to explained variation. R^2 ^describes the goodness of fit - the fraction of sum of squares of all the variables explained by a principal component. For details concerning PCA see Eriksson et al [63]. Outliers were identified using the two powerful methods available in SIMCA-P+: 1) score plots in combination with Hotelling's T2 (identifies strong outliers) and 2) distance to model in X-space (identifies moderate outliers).

## Results

### Neck-shoulder pain

The median intensity and total area sizes of the NSPs' clinical neck-shoulder pain are presented in Table 1. According to the pain drawings, current pain afflicted 5.8% (range 1-27%) of the total body surface, pain at its worst 12.3% (range 3-32) and pain at its least 3.1% (range 0 -14).

**Table 1:** Median and range values for clinical neck-shoulder pain intensity ratings, size of pain areas and different psychological instruments for the trapezius myalgia group (NSP) and for the controls (CON)

	NSP (n = 19) Median (range)	CON (n = 30) Median (range)	p-value
Clinical neck pain intensity (VAS mm)	69 (40-90)	0 (0-20)	<.001
Clinical shoulder pain intensity (VAS mm)	67 (19-88)	0 (0-3)	<.001
Pain duration (months)	120 (36-273)	NA	
Current pain area size (mm^2^)	788 (150-3616)	NA	
Worst pain area size (mm^2^)	1658 (433-4344)	NA	
Least pain area size (mm^2^)	420 (13-1829)	NA	
Awakening problems (KSQ-Aw)	2 (.3-3.3)	1 (0-2.7)	<.001
Sleep disturbances (KSQ-Dst)	2 (1-3.5)	1 (0-3.5)	<.001
Daytime sleepiness (KSQ-Sd)	1.4 (.2-2.6)	.6 (0-2.2)	<.001
Depression (HADS-D)	3 (1-13)	1 (0-5)	<.001
Anxiety (HADS-A)	4 (0-17)	2 (0-10)	.003
Anxiety sensitivity (ASI)	10 (1-33)	8 (2-35)	.225
Pain anxiety (PASS-20)	37 (17-70)	NA	
Catastrophizing (PCS)	15 (6-29)	2 (0-21)	<.001
Fear-avoidance, physical activity (FABQphysical)	10 (4-17)	NA	
Fear-avoidance, work (FABQwork)	22 (0-42)	NA	
Pain disability (PDI)	25 (13-56)	NA	

P-values for differences between groups were derived from Mann-Whitney U-tests. NA denotes not applicable.

Abbreviations: KSQ = Karolinska sleep questionnaire, KSQ-Aw = KSQ- subscale "awakening problems", KSQ-Dst = KSQ- subscale "daytime sleepiness", KSW-Sd = KSQ- subscale "sleep disturbances", HADS = hospital anxiety and depression scale; HADS-D = depression subscale of HADS, HADS-A = anxiety subscale of HADS, ASI = anxiety sensitivity index, PASS = pain anxiety symptoms scale, PCS = pain catastrophizing scale; FABQ = fear-avoidance beliefs questionnaire, FABQphysical = subscale fear-avoidance beliefs for physical activity of FABQ, FABQwork = subscale fear-avoidance beliefs for work of FABQ, PDI = pain disability index.

### Questionnaire scores

NSP generally perceived aspects of their psychological situation, including sleeping problems, significantly worse than CON (Table 1). The only exception was the ASI, where the two groups had similar scores. Even though significant differences existed between NSP and CON the differences were relatively small except for the PCS.

### Pressure pain thresholds

All baseline PPTs over the trapezius and tibialis anterior muscles were significantly lower in NSP compared with CON (Table 2). The differences between groups were smaller for the tibialis anterior than for the trapezius muscles, bilaterally. The mean values for the three PPT points of the right trapezius were 228 kPa (SD 87) in NSP and 450 kPa (SD 117) in CON. The corresponding values for the left trapezius were 232 kPa (SD 78) and 467 kPa (SD 111).

**Table 2:** Median and range values for baseline pressure pain thresholds (PPT, kPa) for the mean of the three sites (T1+T2+T3) in the trapezius muscles and the single site in the midportion of the tibialis anterior muscles (right and left sides) for the trapezius myalgia group (NSP) and for the controls (CON)

		NSP (n = 19) Median (range)	CON (n = 30) Median (range)	p-value
PPT Trapezius (mean kPa)	Right	213 (113-406)	476 (222-600)	<.001
	Left	224 (113-405)	492 (188-600)	<.001
PPT Tibialis (kPa)	Right	567 (140-600)	600 (188-600)	.014
	Left	487 (239-600)	600 (241-600)	.008

P-values for differences between groups were derived from Mann-Whitney U-tests.

### Induced muscle pain

**Figure 1:**
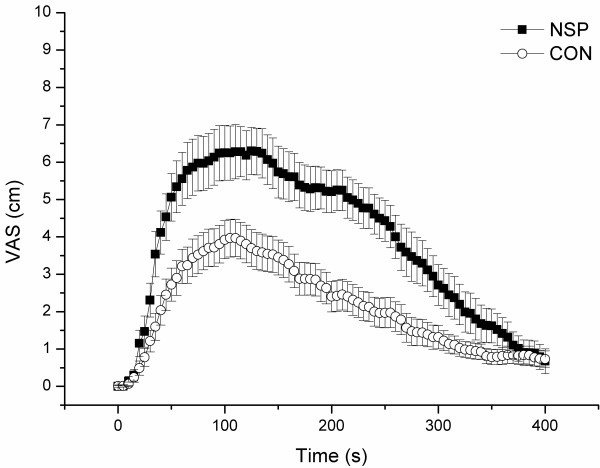
**Pain intensity (VAS, cm; mean ± SD) following hypertonic saline infusion for the trapezius myalgia group (NSP) and for the controls (CON)**.

Hypertonic saline-evoked pain intensity in the tibialis anterior muscle was significantly higher in NSP, both in terms of peak pain intensity, mean pain intensity, and area under VAS curve (Table 3, Figure 1). However, the timing was similar in the two groups as seen in the measurements of pain onset and time to peak pain intensity (Table 3).

**Table 3:** Median and range values for pain intensity ratings on visual analogue scale (VAS 0-10 cm), and total and referred pain drawing area sizes (mm^2^) after hypertonic saline infusion for the trapezius myalgia group (NSP) and for the controls (CON)

	NSP (n = 19) Median (range)	CON (n = 30) Median (range)	p-value
Mean VAS (cm)	4.0 (.4-7.9)	2.8 (.8-7.9)	.002
Area under VAS curve (cm*s)	1616 (126-2842)	758 (50-2501)	.001
Pain onset (s)	25 (0-115)	30 (10-285)	.197
Peak VAS (cm)	7.6 (2.0-10.0)	5.0 (1.0-10.0)	.006
Time to peak (s)	95 (50-350)	120 (35-655)	.335
Total pain area size (mm^2^)	234 (36-3193)	113 (4-864)	.013
Referred pain area size (mm^2^)	65 (0-1376)	0 (0-294)	.020

P-values for differences between groups were derived from Mann-Whitney U-tests.

The pain drawings related to the induced leg muscle pain revealed a significantly larger spreading of pain, both regarding total and referred pain area sizes in the NSP group (Table 3). Proximal spreading of pain was found in four NSP and two CON subjects. These area sizes were 139, 169, 1271, 1834 mm^2 ^and 88, 161 mm^2^, respectively.

### Bivariate correlations in NSP

For NSP self-reported duration of chronic neck-shoulder pain was negatively correlated with baseline PPTs in the right (rho = -.62, p = .004) and left (rho = -.64, p = .003) tibialis anterior muscles, but not with PPTs in the trapezius muscles.

Overall mean PPTs over right and left trapezius were positively correlated with time to pain onset after hypertonic saline infusion (rho = .53, p = .025 and rho = .49, p = .041, respectively) in the NSP group. This suggests that patients with lower thresholds for pressure pain at the site of clinical pain experience a faster onset of hypertonic saline-induced pain in the distant area (i.e., tibialis anterior).

The NSP subjects' mean pain intensity after hypertonic saline infusion was negatively correlated with PPTs in the right (rho = -.56, p = .016) and left (rho = -.53, p = .024) tibialis anterior muscles. Furthermore, the maximum hypertonic saline-induced pain intensity was negatively correlated with PPTs in right tibialis anterior (rho = -.58, p = .013) in NSP. Thus, there seems to be a relationship between pressure pain sensitivity in the lower leg, i.e., an area distant from the site of clinical pain, and the intensity of hypertonic saline-induced pain in the same area.

### Multivariate correlations in NSP

A PCA of NSP yielded a significant model (cumulative R^2 ^= .64) with four principal components (Table 4). Component one (PC1) was dominated by the intercorrelations between different psychological variables from the questionnaire. Hence, PASS-20, HADS-D, ASI, PCS, PDI, FABQwork and all three subscales of the KSQ were significantly and positively correlated (i.e., had the same sign).

**Table 4:** Outcome of the multivariate correlation analysis (principal component analysis; PCA) in the trapezius myalgia group (NSP; n = 19)

		PC1	PC2	PC3	PC4
**Clinical neck-shoulder pain**					
	Current pain area size	0.20	**-0.22**	**0.21**	0.16
	Worst pain area size	0.08	**-0.27**	0.17	**0.29**
	Least pain area size	0.02	0.01	**0.32**	0.02
	Pain duration	0.18	**0.21**	0.02	0.08
	Neck pain intensity	-0.13	**0.30**	-0.17	0.00
	Shoulder pain intensity	0.04	**0.30**	-0.19	-0.12
**Pain after hypertonic saline infusion**					
	Area under VAS curve	0.18	0.13	0.05	**0.30**
	Mean VAS	0.19	0.14	0.10	**0.37**
	Pain onset	-0.09	-0.10	-0.40	0.09
	Peak VAS	0.14	**0.22**	0.12	**0.36**
	Time to peak	0.00	**-0.26**	-0.15	0.04
	Total pain area size	0.13	-0.18	0.04	0.27
	Referred pain area size	0.07	-0.28	0.01	**0.20**
**Pressure pain thresholds**					
	Mean PPT Trapezius right	0.05	-0.21	**-0.30**	0.00
	Mean PPT Trapezius left	0.08	**-0.30**	-0.17	0.00
	Tibialis right	-0.10	**-0.31**	0.07	-0.15
	Tibialis left	-0.15	**-0.25**	-0.03	-0.10
**Psychological factors**					
	KSQ-Aw	**-0.29**	0.02	**0.28**	-0.02
	KSQ-Dst	**-0.27**	0.02	0.23	-0.06
	KSQ-Si	**-0.26**	0.09	0.14	0.02
	HADS-A	-0.20	0.09	-0.28	**0.30**
	HADS-D	**-0.29**	0.06	-0.07	**0.24**
	ASI	**-0.31**	0.06	0.16	0.18
	PASS-20	**-0.28**	-0.12	0.10	0.19
	PCS	**-0.32**	-0.14	-0.15	0.15
	PDI	**-0.20**	0.12	-0.26	**0.26**
	FABQphysical	-0.08	-0.10	0.13	0.18
	FABQwork	**-0.27**	0.01	0.21	-0.13

**Explained variance**	**R^2^**	**0.21**	**0.19**	**0.14**	**0.10**

Variable loadings upon the principal components (PC) in bold indicate variables of significant importance for the component. At the bottom line is given the explained variation (R^2^) of each significant component (PC1-4).

Abbreviations: VAS = visual analogue scale, Abbreviations: KSQ = Karolinska sleep questionnaire, KSQ-Aw = KSQ- subscale "awakening problems", KSQ- Dst = KSQ- subscale "daytime sleepiness"(denoted), KSW-Sd = KSQ- subscale "sleep disturbances", HADS = hospital anxiety and depression scale; HADS-D = depression subscale of HADS, HADS-A = anxiety subscale of HADS, ASI = anxiety sensitivity index, PASS = pain anxiety symptoms scale, PCS = pain catastrophizing scale; FABQ = fear-avoidance beliefs questionnaire, FABQphysical = subscale fear-avoidance beliefs for physical activity of FABQ, FABQwork = subscale fear-avoidance beliefs for work of FABQ, PDI = pain disability index.

The second component (PC2) revealed a negative relationship (i.e., variable loadings with different signs) between chronic pain duration, clinical neck-shoulder pain intensities (VAS) and pain intensity after hypertonic saline infusion (VAS peak) on the one hand and the pain sensitivity measures; PPT tibialis right side and left side, PPT trapezius right side and time to VAS peak after hypertonic saline on the other hand. Thus, a long history of chronic pain and high neck-shoulder pain intensities were associated with low PPTs not only in the area of clinical pain but also in distant pain-free areas. This component also showed a negative relationship between size of clinical pain areas (current and worst) and clinical pain intensities.

Component three (PC3) showed a negative relationship between PPT over the right trapezius and clinical pain areas (current and least) and KSQ-Aw, i.e. individuals with larger areas of clinical pain had lower PPTs over the right trapezius muscle.

The fourth component (PC4) revealed positive relationships between different pain measures and different psychological variables. The clinical pain area size at its worst, area under the VAS curve, mean VAS, peak VAS and the referred pain area after hypertonic saline were all significantly correlated with HADS-D, HADS-A and PDI. Symptoms of anxiety and depression were, thus, associated with increased pain responses to experimental pain induction and a larger spreading of the neck-shoulder pain at its worst.

## Discussion

Major results of the present study were:

• Lower thresholds for pressure pain (PPT) both within the primary clinical pain region and in remote pain free areas were found in NSP.

• The hypertonic saline evoked muscle pain in a remote pain free area was significantly more intense and more locally widespread in NSP than in CON.

• Symptoms of anxiety and depression were associated with increased pain responses to experimental pain induction (i.e., hypertonic saline infusion) and a larger clinical spreading of the neck-shoulder pain at its worst in NSP.

• A long history of chronic pain and high neck-shoulder pain intensities were associated with low PPTs both in the region of clinical pain and in distant pain-free areas. No correlation existed between PPTs and the different psychological aspects.

Previous studies of patients with neck-shoulder pain have reported lower PPTs in painful [9,41,64,65] as well as distant, non-painful muscles [10,17,66,67] when compared to pain-free controls. However, there are also studies reporting no differences in PPTs in the tibialis anterior muscles in this patient group [9,41,64,65]. These contrasting results could be due to differences in patient characteristics. Patients in the current study reported relatively high clinical pain intensities, with a median VAS of 69 mm in the neck and 67 mm in the shoulder region, whereas other studies have reported lower baseline pain intensities (VAS 25-29 mm [64,65]) or (2.4 - 3.6 on 0 - 10 point scales [9,41]) for these regions. Moreover, our patients were allowed to have pain in more than one body region as long as the criteria for widespread pain were not fulfilled; the clinical pain area size at worst afflicted 12% of the total body surface. Consequently, the present study possibly investigated a population with more severe pain than in some of the previous studies [9,41,64,65]. Thus, as proposed by Chien and Sterling [68] it appears that sensory hypersensitivity may represent a continuum of augmented pain processing mechanisms where conditions with greater symptom levels show more profound changes.

The NSP group displayed signs of widespread muscle hypersensitivity to two stimuli - i.e., pressure and chemical stimulation - as evidenced by increased sensitivity to pressure and hypertonic saline induced pain in the tibialis anterior muscle - a site distant to the primary clinical pain in the neck- shoulder region. The increased sensitivity to pressure (PPT) was particularly salient in those patients with a long history of chronic pain, as seen in the correlation between chronic pain duration and PPTs in the tibialis anterior muscles (PC2 in Table 4). This relationship is consistent with the clinical impression of a tendency towards an anatomical spreading of pain with time but has to be confirmed in future prospective studies. Also other studies have shown widespread hyperalgesia in a wide range of chronic pain conditions, including WAD [8,9,18,21], fibromyalgia [19,21,69,70], tension-type headache [16,71], idiopathic neck pain [17], epicondylalgia [14], pelvic pain syndrome [15], and low-back pain [13,72]. Based on studies of chronic WAD [6,9,68,73-76] we recently concluded, that widespread hypersensitivity of pressure can be present without widespread clinical pain [77], which is in agreement with the present results.

Our findings of widespread hypersensitivity of mainly muscle tissue support the suggestions that central pathogenic mechanisms are involved in chronic neck-shoulder pain. Widespread hyperalgesia found in deep tissues has been proposed to occur as a result of sensitization of central nervous system nociceptive pathways or changes in endogenous descending pain modulation mechanisms [18,78]. The widespread alterations of PPT in chronic WAD have been discussed with respect to possible pathophysiological mechanisms: a more or less continuous nociceptive input [79], peripheral nociceptor sensitization [9], secondary hyperalgesia in the primary pain region arising from primary cervical musculoskeletal pathology [79], or a generalized state of hypersensitivity [79]. Several of the proposed peripheral and central mechanisms may be present simultaneously within the primary pain area and consequently be contributing in a complex way to pain and hypersensitivity in this area, whereas widespread spatial alterations reasonably are linked mainly to central mechanisms. However, there are several indications that central alterations in nociceptive processing can be driven by peripheral tissue alterations [76] and peripheral nociceptive input [80,81] also in the chronic stage of a pain condition. Studies using varoius types of blocks indicate a significant peripheral nociceptive input contributing to the alterations in PPT and thermal pain thresholds [80,81].

PPT as well as hypertonic saline induced pain are psychophysical tests and as stated in the introduction can be influenced by different factors e.g., current clinical pain intensity and psychological issues. The relationship to clinical pain intensity and psychological aspects in chronic WAD are either conflicting [6,9] or not present [9,68,73-75,77]. Similarly, there seems to be very little association between psychological variables (i.e., sleeping problems, depression, anxiety, catastrophizing and fear-avoidance beliefs) and PPT in different anatomical regions in the present NSP population. Although it was evident that NSP showed widespread hypersensitivity with respect to PPTs (Table 2), PPTs were more directly connected to the pain intensity per se - the sensory aspects of chronic pain (cf. PC3 in Table 4) - rather than the investigated psychological aspects such as symptoms of anxiety and depression.

In the present study neither the bivariate nor the multivariate analysis revealed correlations between PPTs and psychological aspects. We have recently reported similar results based on multivariate analyses in patients with chronic WAD without widespread clinical pain [77]. Furthermore, we and others have concluded that cold and heat pain thresholds seem to be more strongly correlated with psychological variables and thereby linked to the emotional aspects of pain [5,6,77].

In NSP it was evident that significant correlations existed between pain intensity aspects after induced pain (i.e., intramuscular saline infusion), clinical pain drawing area size and certain psychological aspects (PC4 in Table 4). Hence, more intense symptoms of anxiety and depression together with a higher disability level were associated with increased pain responses to experimental pain induction and a larger area size of the clinical neck-shoulder pain at its worst. These results indicate that the investigated psychological status interacted with the perception, intensity, duration and distribution of induced pain in NSP together with the size of the clinical pain area on the drawing. When interpreting the results from tests of induced muscle pain a bio-psycho-social model is reasonably important. According to the present results muscle pain elicited due to chemical stimuli, but not by pressure, was linked to the psychological status of patients with chronic pain, but this has to be confirmed in other studies. Reasons for these differences between pressure and chemical stimuli can be due to the duration of the nociceptive stimuli during the PPT measurements (parts of seconds) and during the hypertonic saline infusion (several minutes). Interestingly, a study of chronic WAD reported that psychological factors were linked to heat and cold pain thresholds obtained from the skin, while regarding pressure only a very brief nociceptive stimuli is involved [77]. Hence, stimuli duration, and type of stimulus and tissue provoked, may influence the degree of strength between pain threshold and psychological status.

According to the second component (PC2) in Table 4 significant intercorrelations existed among the four pain sensitivity aspects covered in this study; i.e., clinical pain intensities, pain area size, induced pain (peak VAS and time to peak) and PPT (for 3 out of 4 muscles). Hence, high neck-shoulder pain intensities and long clinical pain duration were associated with high peak VAS and a short time to peak VAS after intramuscular saline and low PPTs. Surprisingly, according to the second component, the above mentioned aspects also correlated with small area sizes of clinical pain. Instead, larger pain drawing areas appeared to be associated with increased levels of anxiety and depression according to PC3 and awakening problems in PC3 (Table 4).

### Methodological considerations

With the present study design it was not possible to determine if the sensory hypersensitivity occurred as a direct result of chronic pain or if it was a pre-existing characteristic that predisposes some individuals to develop chronic pain. In some subjects, the local pain area after hypertonic saline infusion expanded to areas where referred pain usually arises in this region. In such conditions, the referred pain area will, by definition, be included in the local pain area, resulting in underestimates of the number of subjects with referred pain.

In the present study we have recruited subjects with chronic neck pain without trauma. We also have related and contrasted our results to studies concerning chronic WAD. However, the question arises if chronic WAD is a separate category compared to other chronic neck pain conditions? There is no consensus in the literature as briefly reviewed by Verhagen and co-workers [82]; the label *mechanical *neck disorders are used with various definitions by some authors. Independent of choice it must be pointed out that categories and diagnoses within this area are not based upon pathophysiological or pathoanatomical mechanisms.

We used the powerful statistical method (principal component analysis; PCA) to investigate the complex multivariate correlation pattern between the psychophysical tests and different psychological variables. Such methods have not been applied in earlier studies investigating the relationships being in focus of this study. The applied multivariate method can handle low subject-to-variables ratios. The PCA implemented in the statistical package used in the present study (i.e., SIMCA-P+) includes, in contrast to other statistical packages (e.g., SPSS), a cross-validation technique in order to achieve stable and valid principal components. However, the multivariate correlation analysis should be viewed mainly as a hypothesis generating method, given the sample size, rather than as a complete model of the interactions between the included variables. To confirm our findings, more experimental research is needed.

## Conclusions

The present study suggests that central sensitization mechanisms are involved in chronic non-traumatic neck-shoulder pain without simultaneous clinical widespread pain since sensory hypersensitivity was found in areas distant to the region of clinical pain. A long history of chronic pain and high neck-shoulder pain intensities were associated with low PPTs both in the region of clinical pain and in distant pain-free areas. Both pressure pain thresholds and chemically induced pain intercorrelated with intensity and area size of the clinical pain. Only the sensitivity to chemically induced pain was associated with the psychological status of the NSP subjects.

## Competing interests

The authors declare that they have no competing interests.

## Authors' contributions

All authors contributed to the design of the study and interpreted data. AS made the basic statistical analyses. The multivariate analyses were made by AS after discussion with BG. AS wrote the first version of the manuscript. All authors critically revised different versions of the manuscript. All authors read and approved the final version of the manuscript.

## Pre-publication history

The pre-publication history for this paper can be accessed here:

http://www.biomedcentral.com/1471-2474/12/230/prepub
